# A Thioester-Containing Protein Controls Dengue Virus Infection in *Aedes aegypti* Through Modulating Immune Response

**DOI:** 10.3389/fimmu.2021.670122

**Published:** 2021-05-13

**Authors:** Shih-Che Weng, Hsing-Han Li, Jian-Chiuan Li, Wei-Liang Liu, Chun-Hong Chen, Shin-Hong Shiao

**Affiliations:** ^1^ Department of Tropical Medicine and Parasitology, College of Medicine, National Taiwan University, Taipei, Taiwan; ^2^ National Institute of Infectious Diseases and Vaccinology, National Health Research Institutes, Miaoli, Taiwan; ^3^ National Mosquito-Borne Diseases Control Research Center, National Health Research Institutes, Miaoli, Taiwan

**Keywords:** *Aedes aegypti*, dengue virus, thioester-containing protein (TEP), innate immunity, transgenic mosquito

## Abstract

Complement-like proteins in arthropods defend against invading pathogens in the early phases of infection. Thioester-containing proteins (TEPs), which exhibit high similarity to mammalian complement C3, are thought to play a key role in the innate immunity of arthropods. We identified and characterized anti-dengue virus (DENV) host factors, in particular complement-like proteins, in the mosquito *Aedes aegypti*. Our results indicate that TEP1 limits DENV infection in *Ae. aegypti.* We showed that *TEP1* transcription is highly induced in mosquitoes following DENV infection. Silencing *TEP1* resulted in the up-regulation of viral RNA and proteins. In addition, the production of infectious virus particles increased in the absence of *TEP1*. We generated a transgenic mosquito line with a TEP1 loss-of-function phenotype under a blood meal-inducible promoter. We showed that viral protein and titers increased in transgenic mosquitoes after an infectious blood meal. Interestingly, expression of transcription factor Rel2 and certain anti-microbial peptides (AMPs) were inhibited in transgenic mosquitoes. Overall, our results suggest that TEP1 regulates the immune response and consequently controls the replication of dengue virus in mosquitoes. This finding provides new insight into the molecular mechanisms of mosquito host factors in the regulation of DENV replication.

## Introduction

Dengue fever is one of the most important arthropod-borne viral diseases. It is caused by four different serotypes of dengue virus (DENV1−4). DENV is a positive-stranded RNA virus that belongs to the *Flaviviridae* family and is transmitted to humans through the bite of infected *Aedes* genus mosquitoes. A current estimate suggests that more than 390 million DENV infections happen worldwide every year (1–3). DENV infection causes a range of symptoms, including undifferentiated fever, dengue fever (DF), and dengue hemorrhagic fever or dengue shock syndrome (DHF/DSS) (2, 4, 5). Dengue is spread through the bite of female mosquitoes, mainly *Aedes aegypti* and, to a lesser extent, *Aedes albopictus*. Mosquitoes acquire the virus when feeding on the blood of an infected person. The virus then replicates within midgut epithelial cells, where it starts to disseminate *via* hemolymph three to five days post-infection (dpi) to infect other tissues, such as fat cells, the trachea, and nervous tissue. Finally, the virus reaches the salivary glands, where it replicates before transmission to another host (6, 7).

Mosquitoes have developed a complex innate immune response system for defense against invading pathogens (6, 8). Complement-like proteins in arthropods function as defense in the early phases of infection (9). Thioester-containing proteins (TEPs), which exhibit high similarity to the mammalian complement C3, are thought to play a key role in the innate immunity of arthropods (10–13). In vertebrates, TEP family members range from broad-spectrum serine protease inhibitors such as α2-macroglobulins to complement factors involved in the recognition and destruction of pathogens (9, 14). An *Anopheles* TEP, induced by *Plasmodium berghei*, was demonstrated to bind and kill ookinetes in the mosquito midgut (13, 15). TEPs in *Anopheles* were shown to play crucial roles in scavenging bacteria *via* phagocytosis (16). It has also been suggested that *Drosophila* TEPs are required for the efficient phagocytosis of Gram-positive or Gram-negative bacteria in S2 cells (17). Additionally, TEPs in the yellow fever mosquito *Ae. aegypti* were identified as key factors for the restriction of flaviviral infections (11, 18). Previous functional studies indicate that TEPs exert potent anti-DENV activity. Some studies also indicate that TEPs may play a role in DENV suppression through the activation of antimicrobial peptides (AMPs) (18). However, the relationship between TEPs and AMPs is still unclear.

AMPs are the effectors of innate immunity in insects and are regulated by a wide variety of signal transduction pathways in response to different microbial infections (6, 8, 19). To date, 17 AMPs have been discovered in the *Ae. aegypti* genome and are categorized into five independent groups: defensins (4), cecropins (10), attacin (1), diptericin (1), and gambicin (1) (19). The mechanisms involved in the regulation of AMPs *via* immune pathways have mainly been studied in *Drosophila* (6, 8, 19). In mosquitoes, Toll, Imd, and JAK-STAT pathways are activated during pathogen infection (6, 8, 19, 20). Pathogenic surface proteins are recognized by immune receptors and trigger downstream transcription factors, such as Rel1 (Toll pathway), Rel2 (Imd pathway), and STAT (JAK-STAT pathway) (21). Then, the activated transcription factors bind to specific regulatory elements for AMP gene transcription initiation (6, 8, 19). The JAK-STAT pathway has been shown to be activated by viral infection in mosquitoes (6, 8, 20). Previous studies also report that AMP expression may be induced by DENV infection in mosquitoes, and AMPs exhibit antiviral activity (6, 8, 18, 20).

In this study, we show that TEP1 limits DENV infection in *Ae. aegypti.* Silencing TEP1 using a reverse genetic approach resulted in an up-regulation of viral RNA and proteins in mosquitoes. In addition, the production of infectious viral particles increased in the absence of TEP1. We generated a midgut-specific TEP1 microRNA (TEP1-miR) expression mosquito with a TEP1 loss-of-function phenotype using the carboxypeptidase (CPA) promoter. We demonstrated that both the viral RNA and titer increased in mosquitoes from this line after an infectious blood meal. Interestingly, transgenic mosquitos with TEP1 loss-of-function inhibited the transcription factor Rel2 of the Imd pathway. Overall, our results suggest that TEP1 regulates the mosquito immune response and consequently controls the replication of dengue virus. These findings provide new insight into the molecular mechanisms of mosquito host factors in the regulation of DENV replication.

## Materials and Methods

### Mosquitoes

UGAL/Rockefeller strain *Ae. aegypti* mosquitoes were kept at 28°C and 70% relative humidity under a light-dark cycle of 12:12 hours as previously described (22, 23). Hatched larvae were transferred to plastic containers with sufficient water and fed with yeast extract daily. Pupae were collected and transferred to a plastic container in an insect dorm. Emerged mosquitoes were fed using cotton balls soaked with a 10% sucrose solution. Female mosquitoes three to five days post- eclosion (PE) were used for our experiments. The sucrose-soaked cotton balls were removed at least 12 hours before blood feeding. Female mosquitoes were permitted to blood-feed on an anesthetized ICR strain mouse for 15 to 30 minutes. ICR strain mice were anesthetized with an intraperitoneal injection of Avertin at a dose of 0.2 mL per 10 g of weight. All animal procedures and experimental protocols were approved by AAALAC-accredited facility, the Committee on the Ethics of Animal Experiments of the National Taiwan University College of Medicine (IACUC approval No: 20200210).

### Cell Culture and Virus

*Ae. albopictus* C6/36 cells were cultured in DMEM/MM (1:1) containing 2% heat-inactivated fetal bovine serum (FBS) and 1× penicillin–streptomycin solution. For virus production, cells were infected with the DENV2 strain 16681 at 0.01 multiplicity of infection (MOI). The culture supernatant was harvested at 7 dpi and stored at −80°C. To determine the viral titer, the virus stock was subjected to examination with a plaque assay, as previously described (24). Approximately 1.0 × 10^7^ PFU/mL DENV2 was used to infect the mosquitoes.

### RNA Extraction and Reverse Transcription (RT)

The whole bodies of three to five mosquitoes or the midguts of 20 to 30 mosquitoes were collected in 1.5 mL tubes containing 0.5 mL Trizol Reagent (Invitrogen). Tissue was homogenized with a rooter-stator homogenizer at room temperature for 5 minutes and centrifuged at 13000 rpm for 10 minutes at 4°C. After centrifugation, the supernatant was transferred to a new micro-tube with 0.1 mL chloroform (J. T. Baker) and mixed thoroughly at room temperature for 3 minutes. Samples were then centrifuged at 13000 rpm for 15 minutes at 4°C and the supernatant was transferred carefully to a new micro-tube with 0.25 isopropanol (J. T. Baker). Samples were gently mixed and stored at −80°C for 30 minutes. After precipitation, the samples were once again centrifuged at 13000 rpm for 30 minutes at 4°C. The supernatant was discarded and 0.5 mL 75% ethanol (Taiwan Burnett International Co., Ltd) was used to wash the RNA pellet. All resulting samples were centrifuged at 8000 rpm for 5 minutes at 4°C and the supernatant was discarded. Finally, the RNA pellet was dried in a laminar flow hood and dissolved in DEPC-H_2_O. After Baseline-ZEROTM DNase (Epicentre) treatment, the RNA sample was stored at −80°C.

The RNA concentration was quantified with a spectrophotometer (Nanodrop 2000, Thermo) and was diluted with DEPC-H_2_O at a concentration of 1 μg/μL. The RNA samples were reverse-transcribed to cDNA with a High-Capacity cDNA Reverse Transcription Kit (Applied Biosystems). The cDNA samples were stored at −20°C for further use. Gene expression was analyzed with a polymerase chain reaction (PCR) using ProTaq Plus DNA Polymerase (Protech). The ribosomal protein S7 gene was used as an internal control.

### Quantitative PCR (qPCR)

The qPCR system used in this study was the SYBR Green dye binding system. SYBR Green binds to the minor groove of DNA and the target gene is quantified by detecting the resulting fluorescence signal. The cDNA sample was quantified with the KAPA SYBR FAST Universal qPCR kit (KAPA) and the qPCR primers were designed using ABI Primer Expression Software. PCR consisted of an initial denaturation at 95°C for 3 minutes, followed by 40 cycles at 94°C for 3 seconds, and 40 seconds at 60°C. Fluorescence readings were measured at 72°C after each cycle. The target gene signal was detected and analyzed with the ABI 7900HT Fast Real-Time PCR System, and relative quantification results were normalized using the ribosomal protein S7 gene as an internal control.

### Double-Stranded RNA (dsRNA) Preparation

RNAi primers were designed with the E-RNAi webservice (http://www.dkfz.de/signaling/e-rnai3//). The T7 promoter sequence (5′- TAATACGACTCACTATAGGG) was incorporated into all forward and reverse RNAi primers. The target gene fragment was amplified with Ex Taq DNA Polymerase (Takara). Fragments were amplified and cloned into a pCR 2.1-TOPO vector at 23°C for 30 minutes using a TOPO TA Cloning Kit (Invitrogen). The constructed plasmid was transformed into HIT-DH5α competent cells. Plasmids from positive colonies were purified using a FarvoPrep Plasmid DNA Extraction Mini Kit (Favogen) and sequenced to confirm that the cDNA was in frame.

The plasmid was digested by a restriction enzyme and fragments were separated using 1% agarose gel. Target fragments were isolated and purified from the gel using a FarvoPrep GEL/PCR Purification Kit (Favogen). The fragments were then amplified with Ex Taq DNA Polymerase (Takara) and purified with the FarvoPrep™ GEL/PCR Purification Kit (Favogen). The purified PCR product was used as the template for synthesizing the dsRNA *in vitro* using a T7-Scribe™ Transcription Kit (Epicentre). The reaction was performed at 37°C for 4 to 12 hours. A solution of 95 μL of DEPC-H_2_O and ammonium acetate (stop solution) was added to stop the reaction and the supernatant was transferred into a new Eppendorf tube with 150 μL of a phenol/chloroform (AMRESCO) solution. Samples were centrifuged at 13000 rpm for 5 minutes at 4°C and the supernatant was transferred to a new Eppendorf tube with 150 μL of chloroform. After another centrifugation at 13000 rpm for 5 minutes at 4°C, the supernatant was transferred to a new Eppendorf tube with 110 μL isopropanol. Samples were gently mixed and stored at −80°C for 30 minutes. Finally, each sample was centrifuged at 13000 rpm for 30 minutes at 4°C. The dsRNA pellets were dried in a laminar flow hood and dissolved in DEPC-H_2_O.

The dsRNA was diluted to a final concentration of 5 μg/μL. Between day three to five post-eclosion (PE), female mosquitoes were injected with 280 nL of dsRNA (5 μg/μL) using a Nanoject II AutoNanoliter Injecter (Drummond Scientific Company). dsRNA against LacZ was used as control dsRNA (dsLacZ). Silencing efficiency was confirmed by collecting the total RNA of mosquitoes three days post-injection for RT-PCR analysis.

### Western Blot Analysis

The whole bodies of three to five mosquitoes or the midguts of 10 to 30 mosquitoes were collected in 1.5 mL Eppendorf tubes containing 100 µL of protein lysis buffer and homogenized with a rooter-stator homogenizer. Each homogenized sample was centrifuged at 13000 rpm for 30 minutes at 4°C and the supernatant was transferred to a QIAshredder column (QIAGEN). The eluted samples were collected and transferred to new Eppendorf tubes at −80°C. The protein concentration was quantified using the Bradford method with a Bio-Rad Protein Assay Dye Reagent (Bio-Rad Laboratories, Inc.). Each protein sample was mixed with the same volume of sample buffer, Laemmli 2× Concentrate (SIGMA), and adjusted to the same volume with 1× sample buffer. To denature proteins for electrophoresis, protein samples were incubated at 98°C for 18 minutes. The protein samples (10 µg in midguts or 60 µg in whole body mosquitoes per lane) were subjected to SDS-PAGE and blotted onto a PVDF membrane (Pall Corporation) for 1.5 hours. The membranes were blocked with 5% skim milk in PBST (1× phosphate-buffered saline, 0.4% tween 20) at room temperature for one hour. Afterwards, the membranes were incubated in the blocking solution with the primary antibody (Anti-NS1, anti-*Anopheles gambiae* TEP1, or Anti-GAPDH) overnight at 4°C. The anti-*Anopheles gambiae* TEP1 antibody used was a gift from Dr. Stephanie Blandin at the Institute of Molecular and Cellular Biology, French National Centre for Scientific Research (CNRS) in Strasbourg, France. Membranes were washed in a PBST solution and incubated with a secondary antibody (anti-rabbit IgG) in the blocking solution at room temperature for one hour. Finally, membranes were washed in PBST and developed using WesternBright Peroxide and ECL (Advansta Inc.) as the substrate for horseradish peroxidase following the manufacturer’s instructions.

### Immunofluorescence Assay

Mosquito midguts were dissected in PBS and fixed in 4% paraformaldehyde (Electron Microscopy, Hatfield, PA) for at least four hours. The fixative was then removed and the midguts were rinsed in PBS, incubated for one hour in 0.1% Triton X-100 in PBS for cell permeabilization, and blocked with a PAT blocking buffer (1% Bovine serum albumin (BSA), 0.5% Triton X-100 in PBS) for one hour. A monoclonal mouse anti-NS1 antibody (YH0023) (Yao-Hong Biotechnology Inc., Taipei, Taiwan) was used as the primary antibody to examine DENV antigens in the midguts. They were then incubated with a 1:500 dilution of goat anti-mouse antibody conjugated with Alexa-488 fluorochrome (Molecular Probes Inc., Eugene, OR). Finally, midguts were mounted with a DAPI-containing medium for confocal microscopy (ZEISS, LSM 510 META Confocal Microscope).

### Plaque Assay

The whole bodies or midguts were collected from TEP1 silenced, dsLacZ-treated, or wild type (control) mosquitoes in 100 μL serum-free medium with antibiotics (penicillin–streptomycin) and stored at −80°C. C6/36 cells were seeded in a 24-well tissue culture plate and incubated at 28°C overnight. The homogenized suspensions of infectious mosquitoes were centrifuged at 18,928 × g for 30 minutes and kept on ice. The cell monolayers were rinsed with PBS and 200 μL of the 10-fold serial dilutions of infectious mosquito suspensions were added for two hours. After viral adsorption, 500 μL 1% methyl cellulose cell media with antibiotics (penicillin–streptomycin) was added and the plates were kept in an incubator at 28°C for five days. The plates were fixed with 4% formaldehyde for one hour at room temperature and stained with 1% crystal violet for 30 minutes. Plaques were quantified *via* manual counting (24).

### Generation of Transgenic Mosquitoes

Female mosquitoes were allowed to lay eggs for 50 minutes three days after the blood meal. The DNA of donor and helper plasmids was mixed at the ratio of 500:300 ng/µL and diluted in a 1× injection buffer (2 mM KCl, 0.1 mM sodium phosphate, pH 6.8). Approximately 500 injected embryos were kept on the filter paper for four days before hatching. Each surviving male and female adult from the injected generation 0 (G0) was outcrossed with three control females or males at a male/female or female/male ratio of 1:3. eGFP fluorescence driven by the 3xp3 promoter manifests at the optic nerve and tracheal gills of G1 transgenic larvae, which were screened with the help of a stereoscopic fluorescent microscope (SZX10, Olympus) (25, 26).

### pMOS1-AeCPA-miR-TEP1-2miR-3xp3-eGFP Vector

The functional stem-loop structure of the artificial mir-based RNAi_TEP1 miRNA was created through the first primer sets, AeTEP1-mir-1-1/Ae-TEP1-mir-1-2 or AeTEP1-mir-2-1/Ae-TEP1-mir-2-2, by PCR. This functional stem-loop miRNA was then extended and flanking sequences with restriction enzyme sites were added with the second primer set, Mir6.1_5′EcoRI/BglII and Mir6.1_3′BamHI/XhoI, to get the precursor TEP1 miRNA unit. The BglII and BamHI restriction enzyme sites of the precursor TEP1 miRNA unit were used for assembling TEP1-miR-1 and TEP1-miR-2 to generate the TEP1-2miRNA cassette (27). Based on the above, following double digestion by the restriction enzymes, EcoRI/BamHI-TEP1-miR-1 and BglII/XhoI-TEP1-miR-2 were integrated concurrently into the EcoRI and XhoI sites of pMOS1_AePUb-Den3-4miR_3xp3-eGFP (GenBank accession: MG603748) to generate a pMOS1_AePUb-miR-TEP1-2miR_3xP3-eGFP transition plasmid with a truncated AePUb promoter (26). The AeCPA promoter was amplified from *Ae. aegypti* genomic DNA by using the following primers: pMOS1_fusion_AeCPA-pr-F and pMOS1_fusion_AeCPA-pr-R (28). Finally, the AeCPA promoter fragments were cloned into the FseI and EcoRI double-digested pMOS1_AePUb-miR-TEP1-2miR_3xp3-eGFP transition plasmid with In-Fusion HD Cloning technology (Clontech), generating the pMOS1-AeCPA-miR-TEP1-2miR-3xp3-eGFP vector.

### Statistical Analysis

All statistical analyses were performed using GraphPad Prism 5 software. Gene expression and fecundity data were analyzed using ANOVA for all independent experiments.

## Results

### An Infectious Blood Meal Activates TEP1 Expression in the Mosquito Midgut

**Figure 1:**
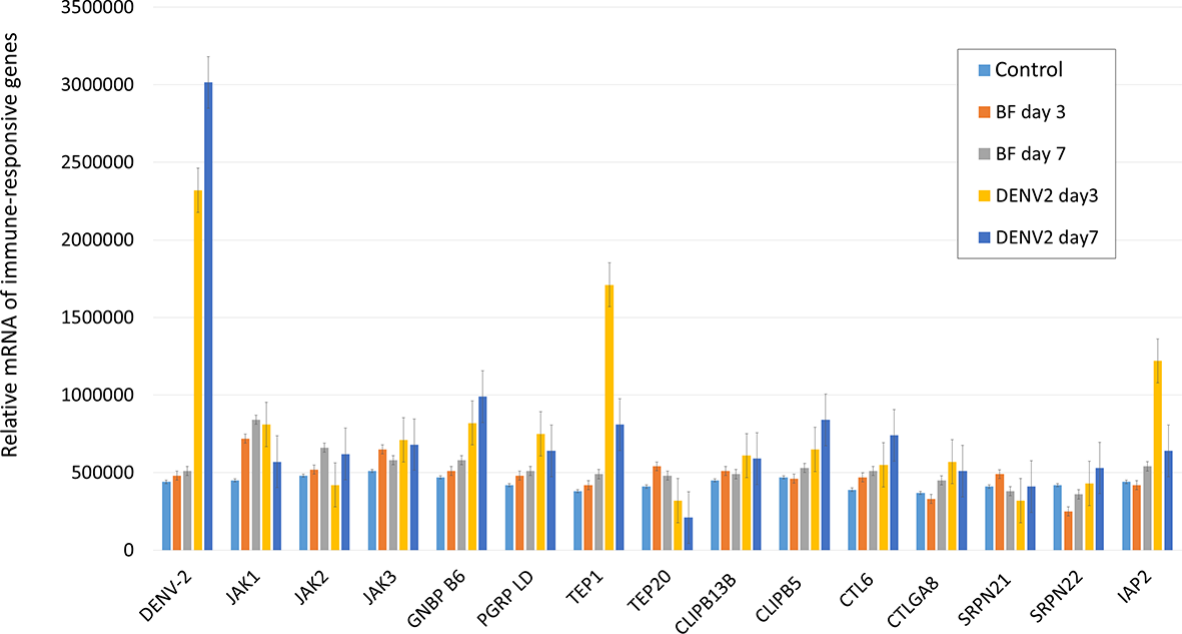
Transcriptional Analysis of Immune-Responsive Genes in the Mosquito Midgut. The relative mRNA levels of the immune-responsive genes from three and seven days after normal (BF) and infectious DENV2 blood-fed (DENV2) mosquito midguts were examined with qRT-PCR analysis. Midguts of mosquitoes fed with non-infectious blood were used as controls. An equal amount of total RNA from each group was used for cDNA synthesis. Ribosomal protein S7 was used for normalization of relative target gene mRNA expression. Values are mean ± S.E. (error bars) of the copy number of each gene. At least three biological cohorts from each time point were used for analysis.

To identify the immune-responsive genes involved in DENV replication in mosquitoes, we selected several immune-responsive genes previously identified as potential inducible genes (29, 30). Total RNA was extracted from the midguts of mosquitoes at three and seven days after an infectious or normal blood meal. The transcriptional profiles of the immune-responsive genes from normal (BF) and infectious DENV2 blood-fed (DENV2) mosquito midguts were examined with qRT-PCR analysis. Interestingly, our results showed that transcription of TEP1 was significantly up-regulated three days post DENV2 infection (**Figure 1**). This indicates that TEP1 is sensitive to DENV infection in the mosquito midgut. Therefore, we investigated the role of TEP1 in DENV2 replication further.

### TEP1 Is Involved in DENV Replication in the Midgut of Mosquitoes

**Figure 2:**
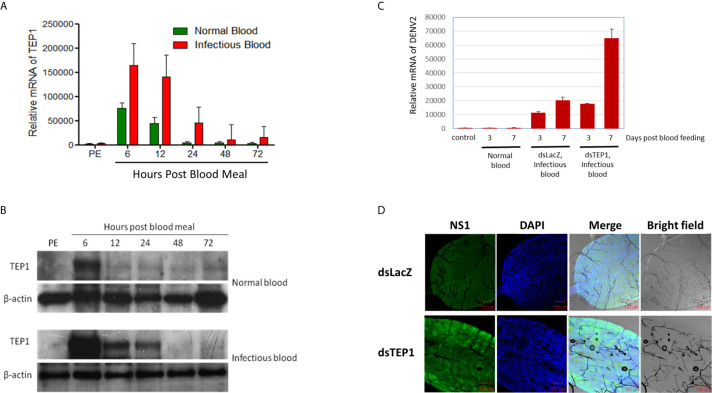
TEP1 Silencing Resulted in an Increase in Viral Load. **(A)** The relative mRNA levels of thioester-containing protein 1 (TEP1) in the midgut of female mosquitoes were quantified by real-time PCR after a normal or dengue virus (DENV) infectious blood meal. Midguts were collected 6, 12, 24, 48, and 72 hours after a normal or infectious blood meal. Equal amounts of total RNA from each group were used for cDNA synthesis. Ribosomal protein S7 was used for normalization of relative target gene mRNA expression. Values are mean ± S.E. (error bars) of the copy number of TEP1. At least three biological cohorts from each time point were used for analysis. **(B)** Midguts were collected 6, 12, 24, 48, and 72 hours after a normal or infectious blood meal. Total protein was extracted and western blot analysis was performed using the polyclonal antibody against *Anopheles gambiae* TEP1. Anti-β-actin antibody was used as the loading control. **(C)** The midguts were collected three or seven days after a naïve blood meal or infectious blood meal. Midguts of 3-day-old, non-blood-fed female mosquitoes were used as controls. Midguts from female mosquitoes were collected and treated with TEP1 double-stranded RNA (dsRNA) three or seven days after an infectious blood meal. Total RNA was extracted and quantified, followed by cDNA synthesis and subjected to quantitative real-time PCR analysis with a specific primer for DENV2. Values are mean ± S.E. (error bars) of the copy number of DENV2. At least three biological cohorts from each time point were used for analysis. Ribosomal protein S7 was used for normalization of relative target gene mRNA expression. **(D)** Mosquitoes pre-treated with TEP1 dsRNA were collected for infectious DENV blood feeding. Mosquito midguts were dissected and collected seven days after feeding with infectious blood for immunofluorescent analysis. The anti-DENV NS1 protein antibody was used to detect the expression of DENV in the midgut of mosquitoes. Alexa Fluor 488 goat anti-mouse IgG was used as a secondary antibody. The images were analyzed by confocal microscopy with single planes presented. Mosquitoes pre-treated with LacZ dsRNA fed infectious blood were used as controls. PE means post-eclosion.

First, we examined the expression profiles of TEP1 between normal and infectious DENV2 blood-fed in mosquito midguts. Midguts from female mosquitoes were collected 6, 12, 24, 48, and 72 hours after a normal or infectious blood meal. Equal amounts of total RNA from each group were used for cDNA synthesis. The transcriptional profiles of TEP1 were examined with qRT-PCR analysis. Our results showed that TEP1 RNA expression was higher in the midgut of the mosquito after a blood meal (**Figure 2A**). The RNA expression level was significantly higher after an infectious blood meal. In order to examine the translational pattern of TEP1, total protein from the midgut of the mosquito was collected 6, 12, 24, 48, and 72 hours after a normal or infectious blood meal. Equal amounts of total protein from each group were used for western blot analysis. Our results show that TEP 1 was activated at 6 hours with a blood medium and decays at 12 hours after feeding, while the expression is higher at 6 hours when the blood includes virus and this was maintained up to 24 hours post infection (**Figure 2B**). To clarify the role of TEP1 in DENV2 replication, we used a reverse genetic approach by introducing TEP1 dsRNA into the mosquito to silence TEP1 expression. The viral genome and NS1 protein expression were activated in the absence of TEP1 (**Figures 2C, D**). These results indicate that TEP1 plays a crucial role in regulating DENV2 replication in the midgut of mosquitoes.

### TEP1 miRNA-Mediated Loss-of-Function Transgenic Mosquitoes Enhance DENV Replication

**Figure 3:**
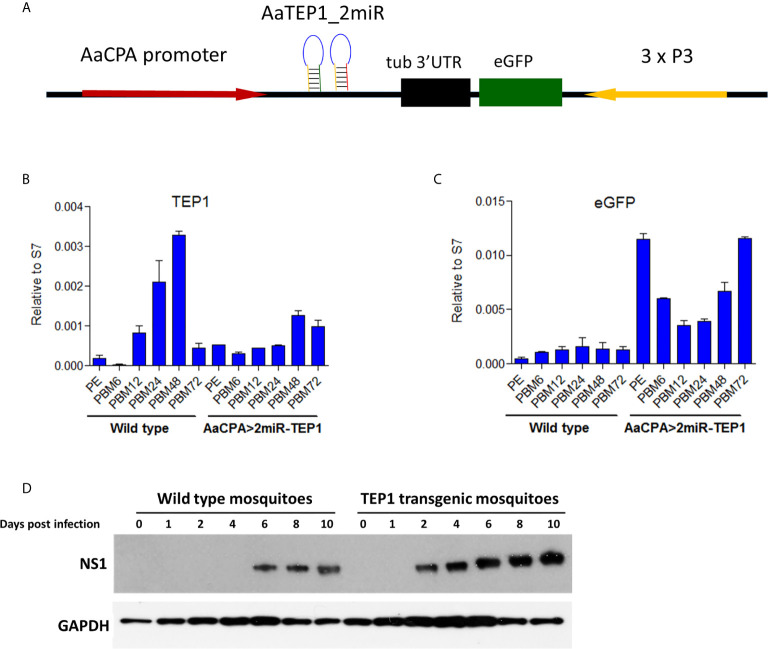
Signal Pathways involved in Dengue Virus (DENV) Replication and Transmission in *Aedes aegypti*. **(A)** Development of a thioester-containing protein 1 (TEP1) loss-of-function transgenic mosquito line with a blood meal-inducible carboxypeptidase (CPA) promoter. Based on the mariner transposon system, the midgut-specific blood meal-inducible CPA promoter (AaCPA) was used for expressing the downstream synthetic miRNAs. AaCPA promoter, *Ae. aegypti* carboxypeptidase A promoter; AaTEP1_2miR, anti-TEP1 miRNA. **(B, C)** miRNA-mediated TEP1 silenced transgenic mosquitoes. The whole bodies of female wild type or transgenic mosquitoes (AaCPA>2miR-TEP1) were collected 6, 12, 24, 48, and 72 hours after a normal blood meal. Total RNA was extracted and quantified, followed by cDNA synthesis and subjected to quantitative real-time PCR analysis with a specific primer for TEP1 **(B)** or GFP **(C)**. Values are mean ± S.E. (error bars) of the ratio of each gene to ribosomal protein S7. At least three biological cohorts from each time point were used for analysis. Ribosomal protein S7 was used for normalization of the relative target gene mRNA expression. **(D)** Anti-DENV phenotype of transgenic mosquitoes. The whole bodies of female mosquitoes were collected 0, 1, 2, 3, 4, 6, 8, and 10 days after an infectious DENV blood meal. Total protein was extracted and western blot analysis was performed with the antibody against DENV NS1 protein. Anti-GAPDH antibody was used as the loading control. PE means post-eclosion.

To investigate the function of TEP1 in mosquitoes, we generated a TEP1 loss-of-function transgenic mosquito using anti-TEP1 miR expression (**Figure 3A**). The expression of anti-TEP1 miR was regulated by a mosquito midgut-specific CPA promoter, which is activated after a blood meal. First, we examined the efficiency of TEP1 silencing in the transgenic mosquitoes. The total RNA of wild type and anti-TEP1 miR-expressed transgenic mosquitoes was collected 6, 12, 24, 48, and 72 hours after a normal blood meal. The expression levels of both TEP1 (**Figure 3B**) and GFP (**Figure 3C**) were determined by qRT-PCR. Compared with wild type mosquito, our results indicate that the TEP1 mRNA level was down-regulated 24 and 48 hours after the blood meal in the transgenic mosquitoes (**Figure 3B**). GFP expression was used as a marker for anti-TEP1 miR-expressed transgenic mosquitoes (**Figure 3C**). To investigate the role of TEP1 in DENV2 replication in the mosquito midgut, replication efficacy in transgenic mosquitoes was examined. Total protein from wild type or anti-TEP1 miR-expressed transgenic mosquitoes were collected 1, 2, 4, 6, 8, and 10 days after a normal or infectious blood meal (**Figure 3D**). Expression of the viral proteins were significantly increased in the transgenic mosquitoes after an infectious blood meal compared to the wild type mosquitoes (**Figure 3D**). Combined, our results suggest that TEP1 is crucial for boosting DENV2 replication in the midgut of mosquitoes.

### TEP1 Silencing Enhances DENV Particle Production in Mosquitoes

**Figure 4:**
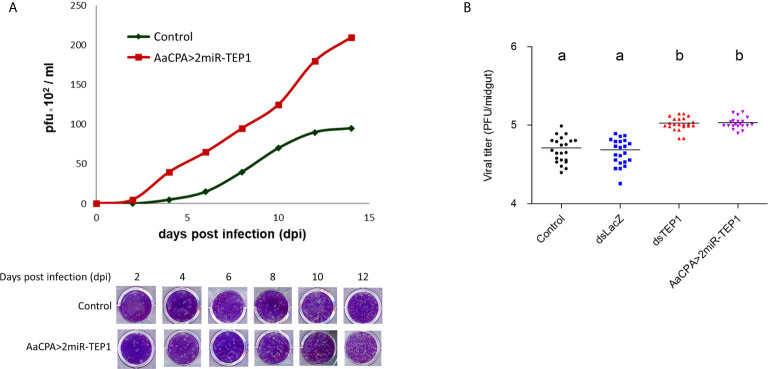
Thioester-Containing Protein 1 (TEP1) Transgenic Mosquito Enhances Dengue Virus (DENV) Infection. **(A)** *Aedes aegypti* control and loss-of-function transgenic (AaCPA>2miR-TEP1) mosquitoes were fed an infectious DENV blood meal. The whole bodies of infected mosquitoes were collected 0, 2, 4, 6, 8, 10, and 12 days after the infectious blood meal and viral titers were determined and quantified with a plaque assay. **(B)** *Ae. aegypti* mosquitoes with silenced TEP1 or mosquitoes from the loss-of-function transgenic line were fed an infectious DENV blood meal. Midguts from infected mosquitoes were collected seven days after the infectious blood meal and the viral titers were determined by plaque assay. Letters indicate the statistical significance according to Tukey’s multiple comparison test. There is no significant difference between them (*p* > 0.05). Control: *Aedes aegypti* UGAL/Rockefeller strain without any transgenic materials or dsRNA treatment.

We examined the effect of TEP1 on infectious virus particle production by comparing the efficiency of particle production between wild type and anti-TEP1 miR-expressed transgenic mosquitoes. Whole body samples of wild type or transgenic mosquitoes were collected 2, 4, 6, 8, 10, and 14 days after an infectious blood meal. They were examined with a plaque assay for infectious virus particle quantification. Our results show that, in response to TEP1 silencing, infectious virus particle production efficiency was higher in transgenic mosquitoes than in wild type mosquitoes after the infectious blood meal (**Figure 4A**). This supports the notion that TEP1 plays a key role in DENV replication. In addition, we examined the effect of TEP1 on infectious virus particle production in the mosquito midgut with a plaque assay (**Figure 4B**). Combined, our results indicate that TEP1 serves as a negative regulator for DENV2 replication in the midgut of mosquitoes.

### TEP1 Silencing Enhances AMP Expression in Mosquitoes

**Figure 5:**
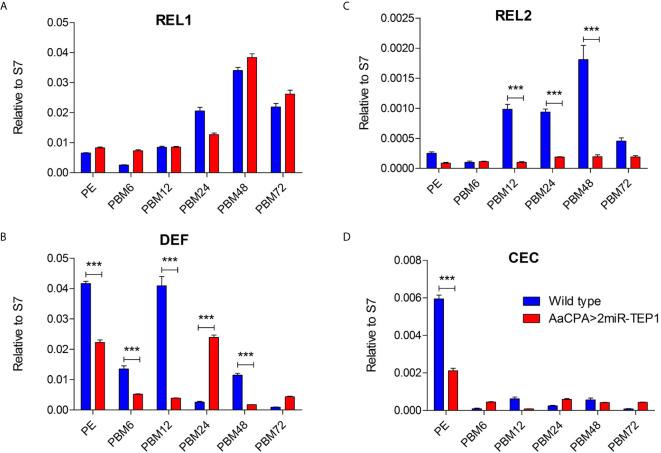
Role of Defensins and Cecropins in Thioester-Containing Protein 1 (TEP1) Transgenic Mosquitoes after Blood Meals. **(A–D)** The whole bodies of female wild type or transgenic mosquitoes (AaCPA>2miR-TEP1) were collected 6, 12, 24, 48, and 72 hours after a normal blood meal. Total RNA was extracted and quantified, followed by cDNA synthesis and subjected to quantitative real-time PCR analysis with a specific primer for Rel1 **(A)**, Rel2 **(B)**, defensins **(C)**, and cecropins **(D)**. At least three biological cohorts from each time point were used for analysis. Ribosomal protein S7 was used for normalization of relative target gene mRNA expression. PE means post-eclosion.

A previous study reported that a TEP-related protein, *Ae. aegypti* macroglobulin complement-related factor (AaMCR), is an essential factor in resisting flaviviral infection in *Ae. aegypti* (18). Moreover, AaMCR interacts with DENV through a homologue of the scavenger receptor-C (AaSR-C), which interacts with DENV and AaMCR. The expression of AMPs is regulated by the AaSR-C/AaMCR complex for potent anti-DENV activity. Additionally, previous studies have also suggested that AMPs play crucial roles in mosquito immune defense against DENV (31–33). Therefore, we hypothesized that TEP1 controls DENV replication by regulating the expression of AMPs. To examine this hypothesis, total RNA was extracted from the midgut of either the wild type or anti-TEP1 miR-expressed transgenic mosquitoes 6, 12, 24, 48, and 72 hours after a normal blood meal. The transcriptional profiles of immune-responsive genes from normal blood-fed mosquitoes were examined with qRT-PCR analysis. Our results showed that Rel1 remained unchanged in TEP1 silenced mosquitoes (**Figure 5A**) whereas Rel2, and defensins were significantly suppressed in TEP1 silenced mosquitoes (**Figures 5B, C**). On the other hand, cecropins was also suppressed at PE stage in TEP1 silenced mosquitoes (**Figure 5D**). Our results point out the crucial role TEP1 plays in controlling immune response in *Ae. aegypti*.

## Discussion

Hosts have developed complex immune systems to defend themselves from invading pathogens before they cause significant physiological damage (6, 8). In mammals, both the innate and adaptive immune systems fight viral infections. In contrast, insects only rely on the innate immune and potentially cytokine-like responses for viral infection defense (6, 8, 15, 30). Without an antibody-based immune response, insects have developed a functional complement-like system to combat pathogens (14, 15). Previous studies on *Anopheles* mosquitoes have shown that TEPs bind to pathogens, such as the malaria parasite *Plasmodium berghei*, and activate phagocytosis to mitigate infection (10, 13, 16). TEP1, TEP3, and TEP4 promote phagocytosis to limit Gram-positive and Gram-negative bacterial infections, and both TEP1 and TEP3 are able to bind to the surface of malaria parasites and activate lysis and melanization (10, 13–16). In *Drosophila*, complement-like protein DmMCR has been shown to bind specifically to the surface of *Candida albicans* to opsonize and promote subsequent phagocytosis (17). TEPs have been identified as key factors in the restriction of flaviviral infections (11, 18). Functional studies indicate that TEPs exert potent anti-DENV activity by regulating AMP activation (18). However, the detailed mechanisms underlying the TEPs-based complement-like system for regulating mosquito immunity against viral infections remain unclear.

More than 30 secreted and membrane-bound proteins have been identified in the human complement network (9, 14). Foreign antigens are recognized by pattern receptors, including C1q, ficolin, and mannose-binding C-type lectin (MBL), which subsequently activate a complement cascade (9). Recent reports have shown that mosquito extracellular C-type lectins, which are the putative homologues of the human MBL, rather than acting as antiviral pattern recognition receptors, act as cellular receptors facilitating West Nile virus and DENV infection (34, 35). We have demonstrated that *Ae. aegypti* TEP1 is highly expressed in the midgut of mosquitoes after an infectious blood meal at both the transcriptional and translational levels. In addition, viral titers were significantly higher in the absence of TEP1. Therefore, these results indicate that TEP1 is an important anti-DENV protein in mosquitoes. We have successfully developed a transgenic mosquito line with TEP1 loss-of-function phenotypes and a blood meal-inducible CPA promoter. The viral titer and RNA in transgenic mosquitoes were higher after an infectious blood meal, further confirming the anti-DENV role of TEP1.

We also demonstrated that TEP1 is an important factor in regulating the replication of DENV. This may be achieved by modulating the expression of AMPs in *Ae. aegypti*. AMPs in insects are effectors of innate immunity, and are regulated by a wide variety of signal transduction pathways in response to different microbial infections (6, 8, 19). To date, 17 AMPs have been discovered in the *Ae. aegypti* genome and they are categorized into five independent groups (defensins, cecropins, attacin, diptericin, and gambicin) (19). The immune pathway mechanisms for AMP regulation have been mainly studied in *Drosophila* (6, 8, 19). In mosquitoes, Toll, Imd, and JAK-STAT pathways are activated upon pathogen infection (6, 8, 19, 20). Pathogenic cell surface proteins are recognized by immune receptors and trigger downstream transcription factors such as Rel1 (Toll pathway), Rel2 (Imd pathway), and STAT (JAK-STAT pathway). Previous studies have evaluated how the expression of AMPs are induced by DENV infection in mosquitoes, and they exhibit antiviral activities (6, 8, 18, 20). We further demonstrated that transgenic mosquitoes with TEP1 loss-of-function inhibited the expression of two AMPs and the transcription factor Rel2 of the Imd pathway. Our results indicated that, compared with wild type mosquitoes, the levels of AMPs expression are suppressed in TEP1-silenced mosquitoes after the mosquitoes take a blood meal. Similar results also descript in previous study. The study shows that mosquito blood meal results in robust activation of the GABAergic system through glutamate-derived GABA production from blood digestion. The enhancement of GABA signaling suppresses antiviral responses, such as AMP induction by the Imd signaling pathway (36). These results suggest that TEP1 play an important role in AMPs production. Our results coincide with previous findings reporting that TEP1 plays an important role in regulating the immune response of mosquitoes.

In conclusion, we demonstrated that TEP1 limits DENV infections in *Ae. aegypti.* Silencing TEP1 using a reverse genetic approach resulted in an up-regulation of viral RNA and proteins in mosquitoes. The production of infectious virus particles increased in the absence of TEP1. Next, we generated a midgut-specific TEP1-miR expression mosquito with a TEP1 loss-of-function phenotype through the CPA promoter. We showed that viral RNA and titer increased in this transgenic mosquito line after an infectious blood meal. Interestingly, transgenic mosquitoes with a TEP1 loss-of-function phenotype inhibited the transcription factor Rel2. Our current results suggest that TEP1 regulates the mosquito immune response and consequently controls DENV replication. These findings provide new insight into the molecular mechanisms of mosquito host factors in regulating DENV replication.

## Data Availability Statement

The datasets presented in this study can be found in online repositories. The names of the repository/repositories and accession number(s) can be found in the article/**Supplementary Material**.

## Ethics Statement

All animal procedures and experimental protocols were approved by the AAALAC-accredited facility, the Committee on the Ethics of Animal Experiments of the National Taiwan University College of Medicine (IACUC approval No: 20200210).

## Author Contributions

S-CW, H-HL, C-HC, and S-HS contributed to conception and design of the study. S-CW, H-HL, J-CL, and W-LL performed the experiments and statistical analysis. S-CW and S-HS wrote the first draft of the manuscript. H-HL, and C-HC wrote sections of the manuscript. All authors contributed to the article and approved the submitted version.

## Funding

This work was supported by the Ministry of Science and Technology (Taiwan) MOST 109-2320-B-002-062-MY3 and MOST 109-2327-B-400-004 to S-HS. This work was also supported by the National Health Research Institutes (Taiwan) 08A1-MRGP12-035 and 09A1-MRGP12-035 to C-HC.

## Conflict of Interest

The authors declare that the research was conducted in the absence of any commercial or financial relationships that could be construed as a potential conflict of interest.
