# Supplementary material for: A Thioester-Containing Protein Controls Dengue Virus Infection in Aedes aegypti Through Modulating Immune Response
Source: Front Immunol. 2021 May 13;12:670122. doi: 10.3389/fimmu.2021.670122 (PMC8155531; doi:10.3389/fimmu.2021.670122)
Supplement: Supplementary file 1 [file DataSheet_1.pdf]

**Table S1. Primers used in this study.**

| | |
|---|---|
| AeTEP1-mir-1-1 | GGCAGCTTACTTAAACTTAATCACAGCCTTTAATGTGAACATCAATTCTACATCTACATAAGTTAATATACCATATC |
| AeTEP1-mir-1-2 | AATAATGATGTTAGGCACTTTAGGTACGAACATCAATTCTACATCTACATAGATATGGTATATTAACTTATGTA |
| AeTEP1-mir-2-1 | GGCAGCTTACTTAAACTTAATCACAGCCTTTAATGTGGAAGGTTCTTACGAAAACACCTAAGTTAATATACCATATC |
| AeTEP1-mir-2-2 | AATAATGATGTTAGGCACTTTAGGTACGGAAGGTTCTTACGAAAACACCTAGATATGGTATATTAACTTAGGTG |
| Mir6.1_5'EcoRI/BglII | GGCGAATTCCGCCAGATCTTTTAAAGTCCACAACTCATCAAGGAAAATGAAAGTCAAAGTTGGCAGCTTACTTAAACTTA |
| Mir6.1_3'BamHI/XhoI | GGCCCTCGAGACGGATCCAAAACGGCATGGTTATTCGTGTGCCAAAAAAAAAAAAAATTAAATAATGATGTTAGGCAC |
| pMOS1_fusion_AeCPA-pr-F | GACGAGATCGGCCGGCCCGCCCTGCAGTATCTTTACATGTAGCTTGTGCATTGAATCC |
| pMOS1_fusion_AeCPA-pr-R | AGATCTGGCCGAATTCGTTGAAATCTCTGTTGAGCAGAAAAAGAAACGAG |
| S7 F | TCAGTGTACAAGAAGCTGACCGGA |
| S7 R | TTCCGCGCGCGCTCACTTATTAGATT |
| DENV F | GAAGACATTGACTGYTGGTGCAA |
| DENV R | CGATGTTTCCACGCCCCTTC |
| JAK1 F | GGATCAAAGCTGCAAAGTCC |
| JAK1 R | GCAATCGTGTAGGCTCGAA |
| JAK2 F | AAAAGATTCGCCCCTCAGAT |
| JAK2 R | ACCTTGCCGTAGTGACCTTG |
| JAK3 F | AATCAAGCGAGCGGAAAAG |
| JAK3 R | AGCGGTTCCTTGCAGAACT |
| GNBP B6 F | TTGCTGTTCTGCGTTGTTGG |
| GNBP B6 R | TGTCAATGTTGGGCGGATGT |
| PGRP LD F | CCGAGCCAAAGTACAACGAT |
| PGRP LD R | TGACGGCACCATTCTGTTTA |
| TEP1 F | CCTGTTCCGTGCAGATTGG |
| TEP1 R | GCAATAAGCGGTCAGCCATAC |
| TEP20 F | TGACTTATTGCATGCTACGCCTAT |
| TEP20 R | CCGAGACCATAACGCTGCTT |

| | |
|---|---|
| CLIPB13B F | GGGATACAAAGCCAAAATGTTCCT |
| CLIPB13B R | AACGGTCTTTCCGCAATCCT |
| CLIPB5 F | TGGTTGGCGTTGCTCAAGTA |
| CLIPB5 R | CCATTCTCCCAGGCGAACTT |
| CTL6 F | GTGGTCGTAGTGCTACAAATTCT |
| CTL6 R | ATTGCTCTGGATGGTGGAGTAT |
| CTLGA8 F | TTCCCCAATTGTTGACGAGGA |
| CTLGA8 R | TCTCCAGGCTTCCAAGAACG |
| SRPN21 F | AACCCAACCCGAAACTGTGT |
| SRPN21 R | AGAGTACTCGGCCTGTAGCA |
| SRPN22 F | GATGGTGCTGCAGAAAGACA |
| SRPN22 R | CGTAAAGCTTGGCGAAAGTC |
| IAP2 F | GGCCAAGGCCGGTTTCTA |
| IAP2 R | TTCACACCAGGCACATTTGAC |
| TEP1 RNAi F | TAATACGACTCACTATAGGGTCACGCATCCACTCTTCTTG |
| TEP1 RNAi R | TAATACGACTCACTATAGGGTCTGGTGCTGCAGGATGTAG |
| EGFP F | ACGACGGCAACTACAAGACC |
| EGFP R | GGGGTGTTCTGCTGGTAGTG |
